# Case of Strangulated Inguinal Hernia,—Adherent Intestine,—Operation and Recovery

**Published:** 1839-04-20

**Authors:** Thomas F. Betton

**Affiliations:** Germantown


// MEDICAL EXAMINER.

DEVOTED TO MEDICINE, SURGERY, AND THE COLLATERAL SCIENCES.

No. 16.] PHILADELPHIA, SATURDAY, APRIL 20, 1839. [Vol. II.

To the Editors of the Medical Examiner.

*Case of Strangulated Inguinal Hernia,—Adherent Intestine,—Operation and Recovery.* By Thomas F. Betton, M. D.

On the 30th of March, ult., I was requested by my friends, Dr. Smith, of Chesnut Hill, and Dr. Johnson, of this village, to visit, with them, Mr. F——, an old gentleman aged seventy-eight, afflicted with strangulated hernia. He had been labouring under all the symptoms of strangulation for ninety-six hours, and all efforts at reduction, attempted by every means which skill could suggest, had proved unavailing. It must be here stated that he had had a rupture for twenty years, which at times gave him uneasiness when at work, but which he could generally relieve by rest, and assuming the recumbent position, and he was, with difficulty, persuaded that this was not, as he supposed. one of his ordinary attacks. His habits throughout his long life had been strictly correct, and his constitution was unimpaired by disease. When convinced of his situation, he submitted cheerfully to the operation, which was performed in the usual manner. On opening the sac, which was perfectly void of fluid, the old hernia was found adherent throughout the whole circumference of the neck; the adhesions were separated and the gut returned. To complicate still further the case, a second hernia was formed below the former, and separated from it by a tendinous band, of about four lines in width. This was strangulated; the stricture was soon found, divided, and the intestine restored. The second hernia was evidently the one that had given rise to the urgent symptoms demanding the operation. Two vessels required securing, the wound was brought together, dressed, and the patient put to bed. He immediately expressed himself much relieved, and, in the course of a few hours, had two or three copious alvine evacuations. His stomach, which previously had rejected even a tea-spoonful of cold water, became tranquil; he drank some tea; took the next day, with great relish, some rye mush and molasses, and was doing as well as possible. From that time he continued to improve, and on dressing the wound on the Tuesday following, we found it united by adhesive inflammation, except in the track of the ligatures. He continued steadily to mend, requiring no medicine, but a dose or two of castor oil, and is now quite well.

The age of the patient, the length of time the gut had been strangulated, the adhesions, and the existence of two herniæ on the same side, render the above case somewhat interesting, and if worthy of a place in your excellent journal, you are at liberty to use it for publication.

*Germantown, April 17th*, 1839.

